# The Prediction of Infectious Diseases: A Bibliometric Analysis

**DOI:** 10.3390/ijerph17176218

**Published:** 2020-08-27

**Authors:** Wenting Yang, Jiantong Zhang, Ruolin Ma

**Affiliations:** 1 School of Economics and Management, Tongji University, Shanghai 200092, China; yangwenting_tongji@163.com (W.Y.); zhangjiantong@tongji.edu.cn (J.Z.); 2 Eli Broad College of Business, Michigan State University, Michigan, MI 48824, USA

**Keywords:** infectious diseases, prediction, bibliometric analysis

## Abstract

Objective: The outbreak of infectious diseases has a negative influence on public health and the economy. The prediction of infectious diseases can effectively control large-scale outbreaks and reduce transmission of epidemics in rapid response to serious public health events. Therefore, experts and scholars are increasingly concerned with the prediction of infectious diseases. However, a knowledge mapping analysis of literature regarding the prediction of infectious diseases using rigorous bibliometric tools, which are supposed to offer further knowledge structure and distribution, has been conducted infrequently. Therefore, we implement a bibliometric analysis about the prediction of infectious diseases to objectively analyze the current status and research hotspots, in order to provide a reference for related researchers. Methods: We viewed “infectious disease*” and “prediction” or “forecasting” as search theme in the core collection of Web of Science from inception to 1 May 2020. We used two effective bibliometric tools, i.e., CiteSpace (Drexel University, Philadelphia, PA, USA) and VOSviewer (Leiden University, Leiden, The Netherlands) to objectively analyze the data of the prediction of infectious disease domain based on related publications, which can be downloaded from the core collection of Web of Science. Then, the leading publications of the prediction of infectious diseases were identified to detect the historical progress based on collaboration analysis, co-citation analysis, and co-occurrence analysis. Results: 1880 documents that met the inclusion criteria were extracted from Web of Science in this study. The number of documents exhibited a growing trend, which can be expressed an increasing number of experts and scholars paying attention to the field year by year. These publications were published in 427 different journals with 11 different document types, and the most frequently studied types were articles 1618 (83%). In addition, as the most productive country, the United States has provided a lot of scientific research achievements in the field of infectious diseases. Conclusion: Our study provides a systematic and objective view of the field, which can be useful for readers to evaluate the characteristics of publications involving the prediction of infectious diseases and for policymakers to take timely scientific responses.

## 1. Introduction

The emergence of infectious diseases is regarded as one of the inevitable public health problems of the world. Although the development of modern technologies and medical science might reduce the negative influence of infectious diseases, the improvement of the transportation system will probably speed up the spread of infectious diseases around the world. For example, the recent outbreak of emerging infectious disease, i.e., severe acute respiratory syndrome coronavirus 2 (later named coronavirus disease 2019, COVID-19) has brought great challenges to the global economy and public health [1,2,3,4]. Therefore, the prediction of the novel or re-emerging infectious diseases can effectively control and prevent large-scale outbreaks and epidemics. The purpose of the prediction is to detect the abnormal distribution of infectious diseases and assess the risk of an epidemic. Meanwhile, the main work of prediction is to collect and analyze information and data about the infectious diseases by using statistical and mathematical approaches, and to explore the temporal and spatial transmission and regular epidemic pattern of infectious diseases [5]. Selecting suitable prediction methods is the premise of implementing the activities about prevention and control of infectious diseases. Not only can it reduce incidence rate and mortality, but also decrease economic and social losses, so as to lay the foundation for the follow-up of relevant policies.

An increasing number of the relevant scholars have studied the prediction of infectious diseases and published many papers in the past two decades [6,7,8]. To provide a comprehensive review of the prediction of infectious diseases, some scholars have proposed systematic reviews, including Racloz et al. [9], Huppert and Katriel [10], Christaki [11], Alessa and Faezipour [12]. However, these reviews mainly focuses on the analysis of the content of the prediction of infectious diseases, such the description of mathematic models or the effects of new techniques, instead of using bibliometric analysis methods to objectively explore developments in the field. Bibliometrics is a cross-disciplinary science of quantitative analysis based on published documents and their references by mathematical and statistical methods [13]. It is extensively performed in research trend detection of infectious disease fields, including dengue [14], Ebola virus disease [15,16], Middle East respiratory syndrome coronavirus (MERS) [17]. However, to the best of our knowledge, there have been few studies on the characteristics and quality of literatures involving prediction of infectious diseases research by using bibliometric methods.

In this paper, we employ two rigorous bibliometric software to comprehensively and objectively analyze the status-quo and the trends of the prediction of infectious diseases based on the academic publications, which are derived from the core collection of the Web of Science (WoS). We firstly analyze the development of prediction of infectious diseases, including the number of publications and citations, the distribution of publication types, co-occurrence network of categories, and co-cited journals by using CiteSpace [18]. Then, VOSviewer is applied to assess and visualize research characteristics from different perspectives, such as co-author, research institutions, countries, keywords, and cited references [19]. Finally, we provide the research guide rules and knowledge structures through the results of bibliometric analysis. It would be convenient for experts and scholars who are interested in the prediction of infectious diseases to rapidly understand their current research status and grasp future research opportunities.

The remainder of this paper is structured as follows. Section 2 briefly describes the data source and methodology of this study. Section 3 explores the development status of the prediction of the infectious diseases domain by using CiteSpace. Section 4 employs the bibliometric analysis technique, i.e., VOSviewer to analyze and visualize the co-authors, research institutions, countries, keywords and references in the prediction of the infectious diseases area. Section 5 discusses the research gaps and promising research opportunities according to bibliometric analysis. Section 6 concludes the paper with the main contributions and presents its limitations.

## 2. Data Source and Methodology

The WoS was published in 1997 and renamed the WoS Core Collection around 2014 [20,21]. In this study, we extracted data from Science Citation Index Expanded (SCIE) and Social Sciences Citation Index (SSCI) from initial publication year to 1 May 2020 in the WoS core collection. In order to improve the quality of retrieval, we employ the advanced search function and the search rules are as follows.

TS = (infectious disease *) AND TS = (‘prediction’ or ‘forecasting’)

Languages = ‘All languages’

Document types = ‘All document types’

Time span = ‘All year’

Database = SCIE, SSCI in WoS Core Collection

Due to the inherent drawbacks of retrieval techniques, we delete the papers with a large deviation (e.g., we only consider the infectious diseases between humans, or zoonotic infections, rather than between animals or plants). After filtering raw literature, 1880 documents met the inclusion criteria, which collected 2876 publications from WoS on 1 May 2020 in text format. The mainly downloaded data are related prediction of infectious diseases domain including titles, author’s information, keywords, references and journals information, etc.

The visualization plays an important role in executing bibliometrics analysis because it can describe the structure and evolution of a certain research field [22]. Nowadays, a lot of visualization tools are available for the convenience of bibliometric research. CiteSpace is a Java-based computer program designed by professor Chen from Drexel University, it is an influential visualization software to obtain quantitative information and discover the related development trends and dynamics in the scientific research field [18,23]. Moreover, VOSviewer is a very effective science mapping tool, which was developed by professors Van Eck and Waltman from the Centre for Science and Technology Studies, University of Leiden [19]. It has been applied in the field of bibliometrics to obtain the visualizing information. Thus, there are a lot of scholars applying CiteSpace and VOSviewer software to perform bibliometrics analysis from different disciplines. For example, Yu, Xu, and Wang [24] used CiteSpace to explore the research trends and patterns among China’s publications in the field of fuzzy theory researches. Song, Zhang, and Dong et al. [25] discovered the current hotspots and potential directions in the publications with the help of CiteSpace. Merigó et al. [26] applied VOSviewer tool to perform bibliometrics analysis to find the citation evolution of the Information Science journal. Yu et al. [27] selected the VOSviewer to identify the historical progress and current situation among the field of support vector machines. According to the above research, both of them are very useful tools in discovering research gaps and detecting the hot topics. In this study, we employ these two analytical tools to explore the status quo and promising research trends in the domain of the prediction of infectious diseases. On the one hand, CiteSpace is used to discover the subject categories and highly cited journals with regard to the prediction of infectious diseases area. On the other hand, VOSviewer is employed to show the core authors, organizations, countries, keywords, and co-cited references in the research field of prediction of infectious diseases.

## 3. The Status Quo of the Field of Prediction of Infectious Diseases

According to the downloaded data in Section 2, the status quo of the prediction of infectious diseases domain can be described, including the development of publications, document types of publications, top journals, and co-occurrence network of categories in this section.

**Figure 1:**
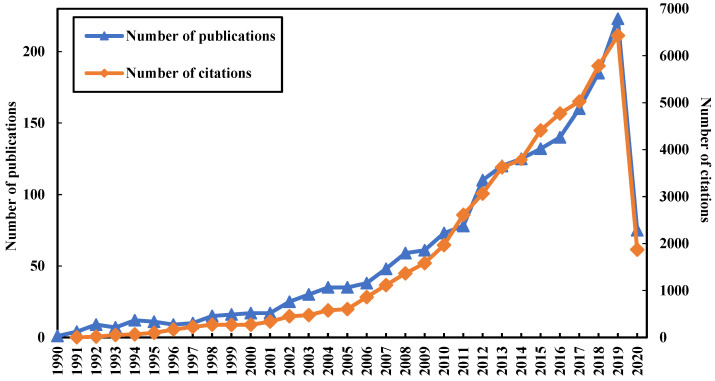
Number of publications and citations in the field of prediction of infectious diseases.

Figure 1 illustrates the evolutionary trajectories of number of publications and citations in the field of prediction of infectious diseases for different years (from 1990 to 2020). From Figure 1, it can be found that the number of publications exhibit a slightly growing trend from 2002 to 2004. This may be because the outbreak of severe acute respiratory syndrome (i.e., SARS) broke out in 2002 and the number of infected people reached a peak value in 2003. In that time, the existence of an epidemic seriously threatened public health and economic stability worldwide. In addition, it is noteworthy that the trend of the number of predictions of infectious disease publications slightly increases from 2011 to 2014 in this figure. The reason might be that the emergence of various infectious diseases, such as MERS, which was defined as a sixth human coronavirus with a high mortality rate in 2012 [28,29]. Thus, an increasing number of experts and researchers focus on the domain after these epidemic emergences. Meanwhile, the emergency of novel and re-emerging infectious diseases have resulted in boosting the scientific discipline each year, leading to a lot of publications in the domain. Thus, it clear from that the overall trends of the number of publications and the number of citations are rising in Figure 1. In addition, the two trends decline from 2019 to 2020 because we only consider the basic information about publications up to 1 May 2020.

**Figure 2:**
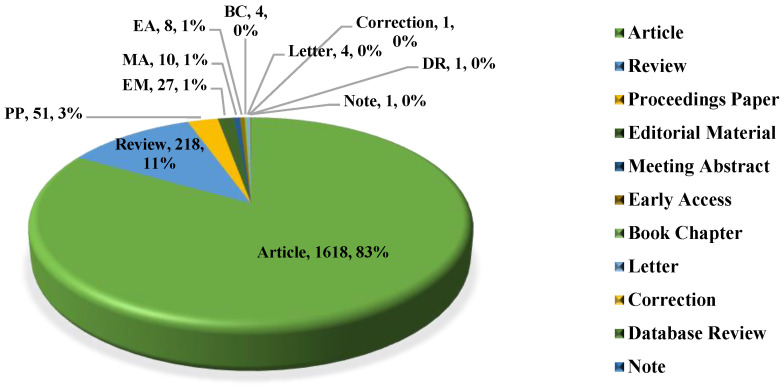
Publications types in the field of prediction of infectious diseases.

Figure 2 displays the distribution of types of the downloaded publications, including article, review, proceedings paper, editorial material, meeting abstract, early access, book chapter, letter, correction, database review, and note. It is obvious that the main types of publications are articles, reviews and proceedings papers (1880, the proportions of the downloaded data are approximately 83%, 11% and 3%) and the remainder of the publications are other types. In addition, it is normal that there are some publications with a small quantity, where the proportion is close to zero, such as a note.

**Figure 3:**
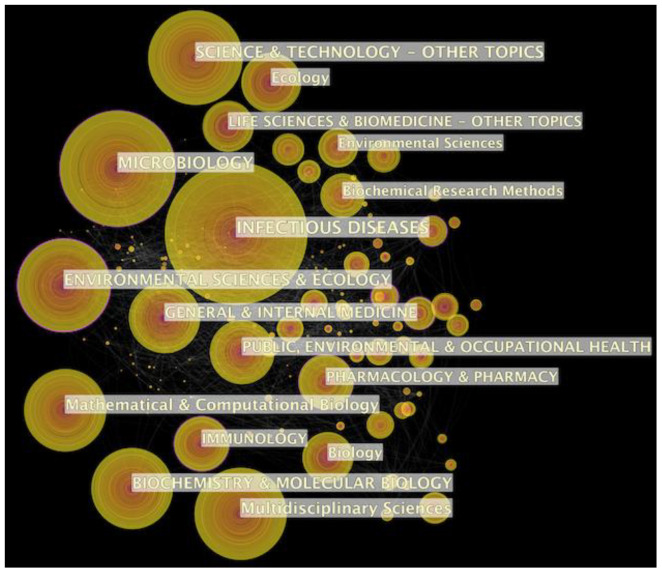
Co-occurrence network of categories in the field of prediction of infectious diseases.

The bibliometrics of subject distribution is a helpful technique to discover the disciplines for the development of a scientific research area [30]. Using CiteSpace software, we can obtain the knowledge mapping result of the co-occurrence network of categories related to the field of the prediction of infectious diseases. Due to the fact that the CiteSpace tool fails to recognize the case of English letters correctly, the visualization result displays some noise. For example, the words “microbiology” and “MICROBIOLOGY” have the same meaning with the different formats, which can cause deviation for the analysis results. Therefore, we should remove the duplicate categories through the built-in function of the software to ensure the reliability and feasibility of the result. After adjusting the outcome, the visualization result is shown in Figure 3. The 1880 publications can be divided into 236 categories. The node size indicates the frequency of the same category, the larger the circle, the higher the classification. And the connection between the nodes indicates the co-occurrence relationship of the two subject categories. It is clearly shown that the top six subject categories are infectious diseases, microbiology, science and technology-other topics, environmental sciences and ecology, multidisciplinary sciences, and mathematical and computational biology in Figure 3. It is no doubt that the prediction of infectious diseases is an interdisciplinary science. Researchers have mainly concentrated on the perspective of epidemiology and bioinformatics to research the prediction of infectious diseases domain. However, it can also integrate with other potential topics, such as pharmacy.

There are 427 different journals that can publish the prediction of infectious diseases literature. It is clear from the result that the top 10 cited journals of the prediction of infectious diseases domain in Table 1. In Table 1, the frequency refers to the total citation number of a certain journal; the WoS categories denotes discipline categories of these journals; the JIF represents the journal impact factor, which is an influential index to measure academic level and paper quality of journals and was obtained from the journal citation reports in 2019; JIF quartile means journal impact factor quartile of various journals, which is employed to assess the publications distribution of an entity, such as a country, institution, research group, or individual, among journals of various fields [31,32]. The finding of this table can show that the top 4 journals are multidisciplinary sciences (i.e., these journals have more comprehensiveness in many domains), the remainder of the journals are mainly related to infectious disease and medicine (i.e., these are professional in some fields). Moreover, all journals are published in the first JIF quartile (Q1 is the best quartile) and most of the cited journals have high impact factors, which shows that these journals are more outstanding to a specific extent. In addition, most of the highly cited journal are from the USA, which shows that the level of American journals about the prediction of infectious diseases domain is still worthy of recognition. Thus, the scholars of the prediction of infectious diseases can study publications and publish related papers with the help of these journals.

## 4. Bibliometric Analysis of the Field of Prediction of Infectious Diseases

In this section, we apply VOSviewer to execute visualization analysis, including collaborative network analysis, keyword co-occurrence analysis, and reference citation analysis in the field of the prediction of infectious diseases.

### 4.1. Collaborative Co-Authors Network Analysis

**Figure 4:**
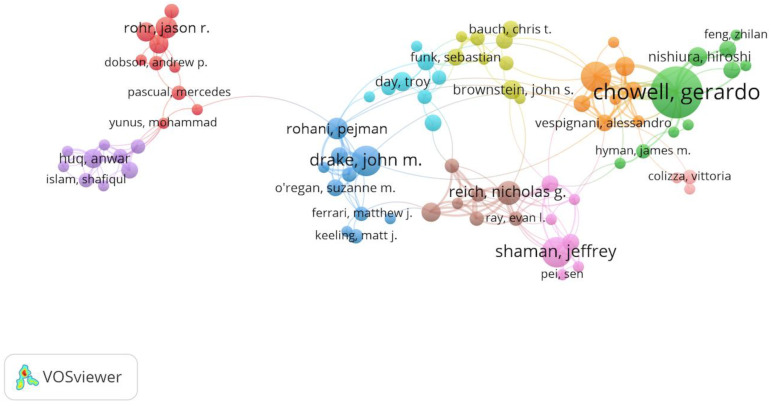
Collaborative network of co-authors in the field of prediction of infectious diseases.

The core author of cooperative network analysis is a useful approach to help corresponding researchers understand the dynamics and updates of critical authors in a certain field. As shown in Figure 4, it shows that the collaborative network of co-authorship in the field of the prediction of infectious diseases with the help of the VOSviewer tool. According to the visualization information, these authors can be classified by different clusters to identify in various colors. In Figure 4, the circle size indicates the frequency of the co-author published documents. The larger the circle, the larger the number of documents is. Additionally, the line connecting two authors suggests a collaborative relationship between them. Based on the outcome of bibliometrics analysis, there are 10 clusters and Chowell is viewed as the largest contributor of co-authors in the prediction of infectious diseases-related area. It is noted that the same color cluster has a strong cooperative relationship between these authors. For example, Shaman is the main core-author in the pink cluster, which has a stronger cooperative relationship than Huq who is in the purple cluster.

### 4.2. Collaborative Countries Network Analysis

**Figure 5:**
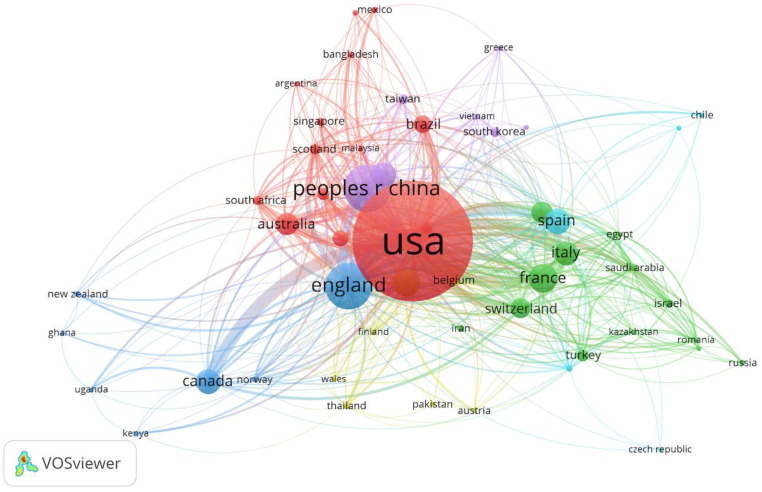
Collaborative network of collaborative countries/regions in the field of the prediction of infectious diseases.

According to downloaded data, the prediction of infectious diseases-related documents were derived from 95 countries and regions with the assistance of VOSviewer software. Similar to Figure 4, the circle size in Figure 5 demonstrates the quantity of publications in a certain country or region. The thickness of line connecting two countries or regions means the relationship strength between them. The thicker the line, the stronger the cooperative relationship. Table 2 shows the top 10 collaborative countries in the field of prediction of infectious diseases, which conveys a similar meaning to Figure 5.

In Table 2, it is worthy of note that the most productive country is the USA with 1880 publications and it has the highest links with other countries or regions in the past few decades. This is might because the scientific research about infectious diseases is related to the outbreak place of epidemic and the research capability of different countries or regions [33]. On the one hand, it is probable that the USA has always been a global scientific leader because of the scale of its economy and the level of its research effort [34,35]. In the USA, there are many high-level national research institutions and they have a good reputation for scientific research. Reputation plays an important role in research effort, thus most of foreign experts and scholars are interested in cooperating with the USA [36]. Actually, the scientific research of China has been growing rapidly in recent years and it has become the largest SCIE papers producer in most situation based on latest references because of the Double First Class Plan in China [37]. Although the document number of China is the top 2 in this study, the total link strength is not high and the impact of China’s publications still lags behind, thus it should improve the quality of scientific research output and strengthen international cooperation in the future [38]. On the other hand, infectious diseases have a negative influence which causes potential risks worldwide to threaten public health and harm the global economy. Therefore, as one of the developed countries, the USA has a high level of scientific research, which promotes international cooperation with other countries [39]. In addition, the interesting finding in Table 2 is that the top 10 cooperative countries have a high-level education. The probable reason is that the country with high-level education can improve the quality of scientific research and promote international cooperation.

### 4.3. Collaborative Institutions Network Analysis

**Figure 6:**
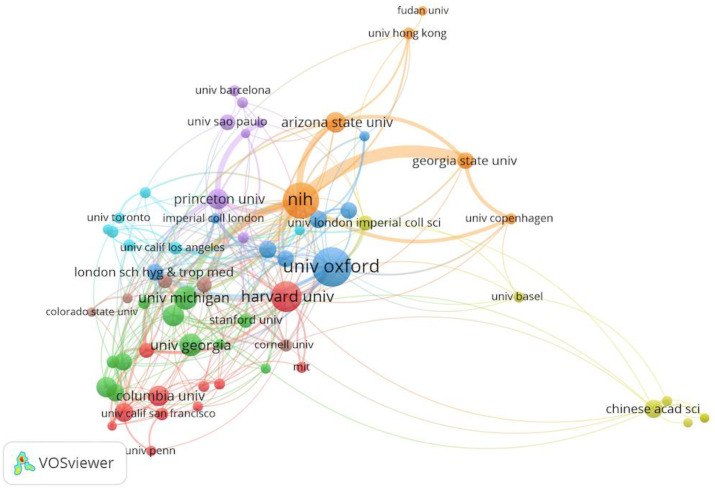
Collaborative network of institutions in the field of the prediction of infectious diseases.

To evaluate the characteristics of collaborative institutions in the field of prediction of infectious diseases, the Figure 6 can be constructed with the help of VOSviewer. From the 2735 institutions extracted from the 1880 publications, 62 important organizations were filtered to discover more useful information by selecting suitable threshold value (i.e., the threshold of the minimum number of documents of an organization is set as 10). In Figure 6, different colors of the circle represent different institutions, the line represents the cooperation strength between research institutions. It clearly shows the result that the USA National Institutes of Health (i.e., NIH) and Georgia State University have the strongest collaboration relationship. Meanwhile, it is easy to find that research institutions belong to the same country with a high cooperation frequency because the communication between these institutions is more convenient. To observe the real situation of published papers among these organizations, we list the top 10 institutions in terms of the prediction of infectious diseases in Table 3. According to Table 3, it can be clearly found that these organizations are mainly from the USA. Therefore, it is further explained that there are many profession scientific research institutions about the prediction of infectious disease domain in the USA. Furthermore, the most productive institution is the USA National Institutes of Health in the USA and it has the highest links with other organizations. This may be because it can provide more real-time infectious disease data for other organizations, so many universities have more interest in cooperating in papers in this field. Besides, University of Oxford, the USA National Institutes of Health, and Harvard University are the core collaborative institution network. In other words, these research institutions provide important assistance to promote the development of the prediction of the infectious diseases domain.

### 4.4. Keyword Co-Occurrence Analysis

**Figure 7:**
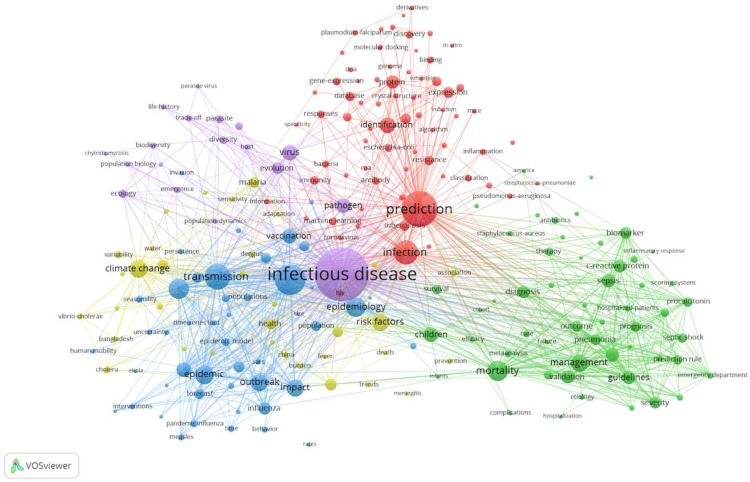
Keyword co-occurrence network about the field of the prediction of infectious diseases.

Keywords play an important role in a publication because this can quickly obtain the basic information about the involved terms, goals, and methods [40,41]. Two or more keywords appear in the same paper at a simultaneous time, which is called keyword co-occurrence [42]. Keyword co-occurrence analysis can identify the research hot topics and monitor the research frontiers transitions of a scientific knowledge domain [30,43,44]. Before performing visualization, keywords should be preprocessed, which should merge different variants with the same meaning to improve the quality of keyword co-occurrence analysis. For example, “infectious-diseases”, “infectious-disease”, “infectious diseases”, “infectious disease” are viewed as the same word. Out of 1880 publications, 227 keywords can be extracted by using the threshold function (minimum number of occurrences of a keyword is 10) of VOSviewer and the network mapping of keyword co-occurrence can be shown in Figure 7. The circle size indicates the total frequency of occurrence for the keywords in the field of the prediction of infectious diseases. The larger the circle size, the more representative the research hotspots and directions in this field [27]. The line indicates the relationship strength between two keywords. In Figure 7, it is clear that these keywords from 1880 publications can be divided into five groups with various colors based on the occurrence relationship. Combined with Figure 3 and Figure 7, it can be found that these groups denote different discipline categories. Specifically, purple groups mainly describe characteristics of infectious diseases, which belong to the subject category of infectious diseases; yellow groups mainly explores the pathogenesis and risk factors for infectious diseases, which can be regarded as life science and biomedicine, and can lay the foundation for the prediction of infectious diseases; blue groups mainly depicts epidemic models for infectious diseases, which can be viewed as mathematical and computational biology and can provide more robust and general models for scientists; the keywords in red groups mainly represents prediction methods and technologies, which can be classified as WoS categories of science and technology and multidisciplinary science; green groups mainly denotes guidelines for infectious diseases, which can be classed as the subject classification of public, environmental and occupational health and can offer effective and reliable countermeasures for infectious disease. These keywords can help corresponding beginners better to search related paper for researching.

To understand co-occurrence of keywords in Figure 7, we list the top 20 keywords in the prediction of infectious diseases in Table 4. Most of the keywords are related to the characteristics of the prediction of infectious diseases, and some special keywords carry interesting information, such as model. In the research of epidemics, the main objective is to reduce the deviations between fitted epidemiological models and the real data based on the dynamics of the spread of infectious diseases, because the difference may important factors that needed to consider in modelling [10]. With the improvement of the infectious disease surveillance system, the quality of observed data has improved, thus it further promotes the development of the predicting and early warning approaches in recent years. According to the dynamic characteristics of infectious diseases transmission, the main objective is to establish a suitable mathematical model to implement qualitative and quantitative analysis and computer simulation of the spread process [5]. Therefore, it is crucial to construct an effective prediction model to rapidly offer follow-up prevention and control measures. For example, the decision-makers have used appropriate mathematical models to successfully control the spread of epidemics during the outbreak of foot and mouth disease in the UK [45,46]. In addition, we can find that “children” also appears in the top 20 keywords. It is not surprising that children are viewed as one of the highly contagious populations. Therefore, there are many factors that can be considered based on the real situation by many experts and researchers, such as age, population density, human behaviors, climate-change, to improve the precision of prediction in the model.

### 4.5. Reference Co-Citation and Highly Cited Paper Analysis

**Figure 8:**
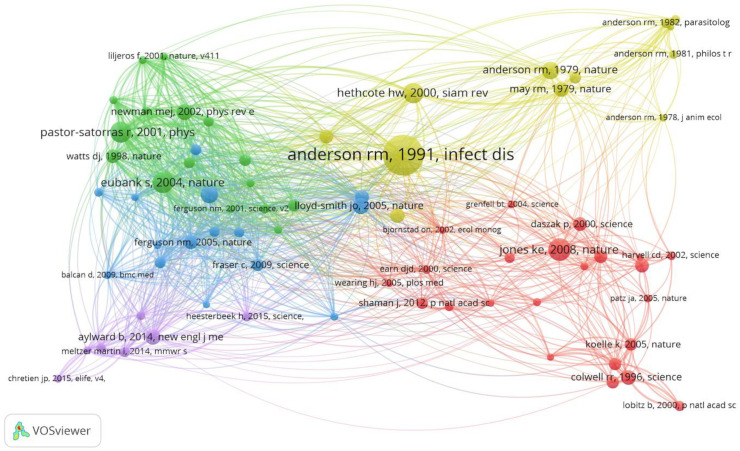
Co-cited reference network in the field of prediction of infectious diseases.

Reference co-citation analysis was used to described two documents co-occurrence in the references list of another publication [47]. The co-citation analysis of reference is critical to capture the development trends and find research opportunities for related scholars and researchers in a certain area [24]. To discover the reference co-citation relationship in the field of the prediction of infectious diseases, the VOSviewer tool can be employed to visualize as shown in Figure 8. Out of 71,690 cited references, there are 111 papers over 15 times co-cited by these publications in Figure 8. The circle size represents the frequency of co-cited references, the connection between two circles represents the relationship of references. In Figure 8, it is clear that Anderson [48] is the largest circle because it belongs to the most cited paper up to now in the field of the prediction of infectious diseases. That is to say, the paper made a great contribution to the domain. To improve the readability of Figure 8, we construct Table 5 to describe the top 10 most-cited papers in the field of prediction of infectious diseases.

According to the collected data, the top 10 cited publications were shown in Table 5 from their initial publication year until 1 May 2020, in the field of prediction of infectious diseases. Based on Table 4, some interesting information can be discovered. After reading these papers, we can find that many papers are co-authored. It is probable that the problem is hard to solve, which needs the wisdom of many experts and scholars in the field of prediction of infectious diseases. In addition, it is obvious that most of these highly cited papers have a high JIF respectively. It also verifies that the highly cited papers have a strong relationship between the impact factors, which can help keep the quality of the journal [49,50,51].

Generally speaking, proceedings papers related about the prediction of infectious diseases of reflecting leading edge research and contemporary perspectives. Therefore, it could be very interesting to conduct a comparative analysis between two types of literature, journal articles and conference papers, to further obtain understanding of the infectious diseases field. In Table 6, we list the top five cited conference papers to perform a comparative analysis with Table 5. Comparing Table 5 with Table 6, it can be found that conference papers are less cited than journal papers. This may be because journal papers are more formal than conference papers to a certain extent. In addition, these high-cited journal papers and proceedings papers were published by developed countries. It is not surprising that developed countries pay more attention to education and scientific research. The predictions are made by infectious diseases-related researchers who are interested in studying these highly cited papers to enhance relevant academic knowledge.

## 5. Research Gaps and Future Research Directions

In this section, we discuss research gaps and some promising scientific direction for future research in the prediction of infectious disease field, which can be shown as follows:

- The field of prediction of infectious diseases can be viewed as interdisciplinary. In Figure 3, the subject categories mainly focus on epidemiology and bioinformatics about the prediction of infectious diseases-related fields. It is likely that these categories can better explore the characteristic and pathogenesis of epidemic for infectious diseases. Moreover, the category of mathematical and computational biology is play an important role in this field. It provides more mathematical models and methods to improve the precision of prediction, which is the focus of further ongoing research. In addition, scholars and experts can explore new categories from other perspectives to acquire more meaningful conclusions, such as psychology. Although this category does not appear in Figure 3, psychology may be a potential research opportunity in the future. For example, Weston, Hauck, and Amlôt [66] discuss the role that the social-psychological literature concerning the health behaviors and behavioral change in the description of the infectious diseases models, such as emotional responses and social distancing. These psychological elements can provide new angles to prevent and control the transmission of infectious diseases. Thus, how to excavate new categories with different backgrounds to analyze and discuss the prediction of infectious diseases can be explored in the future.
- Emerging technologies provide many opportunities for improving the precision of infectious diseases prediction. According to Figure 3, it can be found science and technology-other topics is the top 3 subject category. Recently, some studies have discussed the emerging technologies to predict infectious diseases, including remote sensing technology [67], artificial intelligence [68], big data analysis [69,70], social media [71]. The emergence of new technologies is important for researches and scholars to improve the prediction precision of infectious diseases. As a result, how to find more effective and reliable new techniques to rapid responses the demands of the prediction can be explored in the future.
- There are many factors that should be considered in prediction of infectious diseases. Based on Table 4, we can find that children (i.e., age) and climate change are high-frequency keyword co-occurrence, and these may be pathogenic factors. There are some factors often discussed in the prediction models of infectious diseases, including human behaviors, temperature variation, population mobility, the relationship between humans and wildlife, etc. [52,71,72,73]. However, some new factors have also been considered in the model recently. For example, most of the papers considered individual behavioral into prediction models [74,75], some of them employed social network to estimate the transmission of infectious diseases [76]; a substantial proportion of the papers integrated economic or game-theoretic factors into infectious diseases models [77,78]. It is important for scientists to select suitable factors for improving the reliability and validity of the prediction in the prediction models. Consequently, how to choose reasonable scientific factors in models for different infectious diseases can be focused on in the future.
- According to our analysis, current research about the prediction of infectious diseases mainly concentrate on mathematical models to describe the dynamics of epidemics and the influence of transmission from Figure 7. However, different prediction model of infectious diseases may have different effects. Therefore, it is necessary to propose a more robust and general mechanism for different infectious diseases. Combining different approaches can be viewed as an interesting direction, such as quantitative modelling [79], simulations [80] and empirical research [81,82] to further enhance the precision and effectiveness of models. Therefore, how to use different methodologies to discover more meaningful results in the prediction of infectious diseases can be detected in the future.

## 6. Conclusions

During the past few decades, there have been many reviews about the prediction of infectious diseases, but no in-depth scientometrics analysis. In this paper, a bibliometric study of the prediction of the infectious diseases domain is presented, which objectively analyzes the current status and research hotspots from the perspective of bibliometrics with the help of CiteSpace and VOSviewer visualization software. Based on the downloaded data from WoS core collection, we obtain 1880 related publications in the field of prediction of infectious diseases. To find more meaningful results, we implement the bibliometric analysis, including collaborative network analysis, co-citation analysis, and co-occurrence analysis.

According to the above bibliometrics analysis, the main conclusions of this study can be summarized as follows: (1) the outputs of publications have substantially increased about the prediction of infectious diseases domain from 2002 to 2004 due to the breakout of SARS; subsequently, other epidemics broke out have promoted the increase of publications; (2) there are 11 different documents types about the prediction of infectious diseases, in which around 83% documents published in articles; (3) *Nature* is the most popular journal for researchers and scholars in the field of prediction of infectious diseases and most of the highly cited journals have high impact factors, and it is further verified that there is a relationship between journal and impact factor; (4) visualization result shows that the cooperation of countries in the field of prediction of infectious diseases is more and more extensive and international. The USA is the most productive country, as well as countries with high levels of education, have a wealth of research scientific results about the prediction of infectious diseases; (5) the most influential institutions are University of Oxford, the USA National Institutes of Health and Harvard University, and the US National Institutes of Health (i.e., NIH) and Georgia State University have most cooperative relationships. In addition, it is found that most of the organizations belong to the same country with a high cooperation frequency; (6) the most frequently cited work in prediction of infectious diseases domain is Anderson et al. [48] and most of these highly-cited papers have a high IF. It also verifies that the relationship between highly cited papers and the impact factors. Above all, it is beneficial for some scholars who are interested in the field of prediction of infectious diseases to gain basic knowledge and grasp the research characteristic in their future works. In addition, the global outbreak of COVID-19 has inflicted great harm on public health and social development, which attract many scholar’s attention recently [83,84]. The experiences and methods of prediction of infectious diseases can offer scientific research basis and guidelines for COVID-19.

In this study, there are some limitations that should be considered in the future. We only employ data from the database of SSCI and SCIE in the WoS core collection to search for the prediction of infectious diseases, which may lead to less perfect results of bibliometric analysis. Furthermore, the number of prediction of infectious diseases-related publications may increase in 2020 because new documents will be published in this year. Thus, we will increase other databases, such as Scopus, Google Scholar and PubMed to conduct a perfect comparative analysis between journal articles and conference papers to obtain a thorough understanding of the prediction of infectious diseases domain, and then we will implement a complete annual bibliometric analysis in a future study. In addition, we will consider a comprehensive analysis of the highly cited publications for their contents to further detect the evolution of the topical areas and provide new insights instead of the current narrower scope that is based on mixed documents of prediction of infectious diseases-related.

## Figures and Tables

**Table 1:** The top 10 cited journals in the field of prediction of infectious diseases (up to 1 May 2020).

Rank	Journal	Frequency	WoS Categories	JIF	JIF Quartile	Country
1	*Nature*	781	Multidisciplinary Sciences	42.778	Q1	UK
2	*Proceedings of the National Academy of Sciences of the United States of America*	764	Multidisciplinary Sciences	9.412	Q1	USA
3	*PLoS ONE*	707	Multidisciplinary Sciences	2.74	Q1	USA
4	*Science*	689	Multidisciplinary Sciences	41.845	Q1	USA
5	*New England Journal of Medicine*	568	Medicine, General & Internal	74.699	Q1	USA
6	*Lancet*	526	Medicine	60.392	Q1	UK
7	*Clinical Infectious Diseases*	435	Immunology, Infectious Disease, Microbiology	8.313	Q1	USA
8	*Emerging Infectious Diseases*	359	Immunology, Infectious Disease, Microbiology	6.259	Q1	USA
9	*Journal of Infectious Diseases*	310	Immunology, Infectious Disease, Microbiology	5.022	Q1	USA
10	*Proceedings of the Royal Society B-biological Sciences*	307	Ecology, Evolutionary Biology	4.637	Q1	UK

WoS–Web of Science; JIF–journal impact factor.

**Table 2:** The top 10 collaborative countries/regions in the field of the prediction of infectious diseases.

Rank	Country	Documents	Total Link Strength
1	USA	749	553
2	People Republic of China	215	130
3	England	209	281
4	France	113	177
5	Germany	105	127
6	Spain	94	125
7	Canada	93	101
8	Japan	90	94
9	Italy	88	128
10	Australia	81	121

**Table 3:** The top 10 collaborative institutions in the field of the prediction of infectious diseases.

Rank	Institution	Documents	Total Link Strength	Country
1	University of Oxford	44	35	UK
2	The USA National Institutes of Health	40	81	USA
3	Harvard University	34	33	USA
4	The University of Michigan	26	24	USA
5	The University of Georgia	25	11	USA
6	Emory University	23	19	USA
7	University of Maryland	22	13	USA
8	Princeton University	22	35	USA
9	Columbia University	22	10	USA
10	Arizona State University	22	24	USA

**Table 4:** The top 20 keywords about the field of the prediction of infectious diseases.

Rank	Keywords	Occurrences	Rank	Keywords	Occurrences
1	Infectious disease	592	11	Management	97
2	Prediction	307	12	Risk factors	95
3	Model	238	13	Impact	92
4	Transmission	181	14	Outbreak	91
5	Infection	167	15	Virus	81
6	Dynamics	126	16	Identification	80
7	Mortality	121	17	Vaccination	75
8	Epidemiology	116	18	Spread	74
9	Epidemic	108	19	Pathogen	74
10	Climate change	104	20	Children	72

**Table 5:** The top 10 cited publications in the field of prediction of infectious diseases.

Rank	Author/Year	Title	ST	CN	JIF
1	Anderson et al. [48] (1991)	Infectious diseases of humans: dynamics and control	*Oxford University Press*	559	
2	Jones et al. [52] (2008)	Global trends in emerging infectious diseases	*Nature*	404	43.07
3	Eubank et al. [53] (2004)	Modelling disease outbreaks in realistic urban social networks	*Nature*	370	43.07
4	Pastor-Satorras et al. [54] (2001)	Epidemic spreading in scale-free networks	*Physical Review Letters*	319	8.385
5	Hethcote [55] (2000)	The Mathematics of Infectious Diseases	*SIAM Review*	204	11.431
6	Anderson et al. [56] (1979)	Population biology of infectious diseases	*Nature*	185	43.07
7	Mossong et al. [57] (2008)	Social Contacts and Mixing Patterns Relevant to the Spread of Infectious Diseases	*Plos Medicine*	171	10.5
8	Lloyd-Smith et al. [58] (2005)	Superspreading and the effect of individual variation on disease emergence	*Nature*	165	43.07
9	Newman [59] (2002)	The spread of epidemic disease on networks	*Physical Review E*	150	2.296
10	Aylward et al. [60] (2014)	Ebola Virus Disease in West Africa-The First 9 Months of the Epidemic and Forward Projections	*New England Journal of Medicine*	136	74.699

**Note**: In Table 5, ST means source title, which is the published journal of the paper; CN means the citation numbers, which is the total citation times of a certain paper; JIF–journal impact factor.

**Table 6:** The top 5 cited proceedings papers in the field of prediction of infectious diseases.

Rank	Author/Year	Title	MN	CN	CP
1	Cardinal et al. [61] (1996)	On the application of integer-valued time series models for the analysis of disease incidence	*Annual Meeting of the Society-for-Epidemiological-Research*	43	USA
2	Gavin et al. [62] (2004)	Fuzzy expert systems and GIS for cholera health risk prediction in southern Africa. International Symposium on Environment Software System	*International Symposium on Environment Software System*	40	USA
3	Schroeder et al. [63] (2005)	GIS, geostatistics, metadata banking, and tree-based models for data analysis and mapping in environmental monitoring and epidemiology	*International Potsdam Symposium on Tick-Borne Diseases*	36	Germany
4	Jewell [64] (2007)	A novel approach to real-time risk prediction for emerging infectious diseases: a case study in Avian influenza H5N1	*GisVet 2007 Conference*	30	Denmark
5	Kelly et al. [65] (2015)	One health proof of concept: bringing a transdisciplinary approach to surveillance for zoonotic viruses at the human-wild animal interface	*Symposium of the International society-for-Veterinary-Epidemiology-and-Economics*	27	Mexico

**Note**: In Table 6, MN means meeting names; CN means the citation numbers, which is the total citation times of a certain paper; CP means conference place.
